# Discovery of the function of the heart and circulation of blood

**Published:** 2009-05

**Authors:** Gerald Friedland

**Affiliations:** Professor Emeritus, Department of Radiology, Stanford University School of Medicine, USA

In *Medicine’s 10 Greatest Discoveries*, which I co-authored with cardiologist Meyer Friedman, we stated that William Harvey’s discovery of the function of the heart and the circulation of blood was the greatest medical discovery of all time. Not only did it initiate the field of physiology, but it also introduced the principle of experimentation in medicine.

Long before Harvey, Galen, born on 9 September AD 129 in Pergamon, Greece, discovered the pulmonary circulation. In AD 157, at the age of 28, he was chief physician to the gladiators in Pergamon, where he watched the still-beating hearts of fighters who lay dying, their chests ripped open by their opponents’ blades. Later, when he moved to Rome, he carried out vivisections on monkeys and pigs, and again observed the pulmonary circulation. But he erroneously thought that arterial blood originated in the heart and venous blood in the liver, and that the liver pumped blood to the rest of the body where it was consumed.

Michael Servitus, a Spanish physician born in 1511, possessed a thorough knowledge of Galen’s writings. He carried out vivisections on animals and also observed the pulmonary circulation. He was burned at the stake for his religious beliefs on 27 October 1553.

Realdo Columbo (1515–1559) confirmed the pulmonary circulation on vivisection. He also discovered that the heart’s four valves permitted flow of blood in one direction only: from the right ventricle to the lungs, back to the left ventricle, and from there to the aorta.

William Harvey was born on 1 April 1578. At the age of 16, he was awarded a medical scholarship and graduated from Cambridge University in 1597 with a Bachelor of Arts degree. One of his professors encouraged him to travel to Padua, Italy, where he studied under Hieronymus Fabricius, who had discovered the valves in veins. After he returned to London, he married the daughter of a prominent physician, and became a Fellow of the Royal College of Physicians of London.

Harvey performed vivisections on dogs and invited Fellows of the Royal College of Physicians of London to attend, so that they could verify his findings. He estimated that the capacity of the heart was 43 g, that about 6 g of blood went through the heart every time it pumped, and that the heart beats 1 000 times every half hour. This means that it pumps about 5 kg of blood in a half hour, or about 245 kg in a day. This proved the theory that the body consumed blood was incorrect.

Harvey also proved that blood flows in two separate loops, the pulmonary circulation and the systemic circulation. He then tied a ligature on the upper arm of a person, which distended the veins in the forearm and made the valves of the forearm clearly visible. He tried to force blood in a vein down the forearm, but to no avail. When he tried to push it up the arm, it moved easily. Harvey had proved that the venous blood flowed to the heart, and that the body’s valves in the veins maintained the one-way flow.

Harvey published a book on his discoveries in Latin in 1628, with the title *De motu Cordis et Sanguinis in Animalibus* and a second book in English in 1653, titled *The Anatomical Exercises of Doctor William Harvey Professor of Physic and Physician to the King’s Majesty, Concerning the Motion of the Heart and Blood.*

Harvey also believed in witches. At the request of King Charles I, he gladly agreed to examine a suspected witch. Harvey also frequently remarked that ‘Europeans know not how to order or govern their women’ and that ‘Turks are the only people who use them wisely’. The man was not a feminist!

